# Supplementary material for: Meat Intake, Cooking Methods, Doneness Preferences and Risk of Gastric Adenocarcinoma in the MCC-Spain Study
Source: Nutrients. 2022 Nov 16;14(22):4852. doi: 10.3390/nu14224852 (PMC9695943; doi:10.3390/nu14224852)
Supplement: Supplementary file 1 [file nutrients-14-04852-s001.zip › nutrients-1974891-supplementary.pdf]

Table S1. Sensitivity analysis to explore the role of *Helicobacter pylori* (HP) infection in the association between gastric adenocarcinoma incidence and total, white, red and processed meat consumption.

		**Model 1[a]**	**n=3279**				**Model 2[b]**	**n=3092**				**Model 3[c]**	**n=1979**				**Model 4[d]**	**n=1979**				**Model 5[e]**	**n=1760**			
	**Sv/week**	**Controls**	**Cases**	**OR[f]**	**LL[f]**	**UL[f]**	**Controls**	**Cases**	**OR[f]**	**LL[f]**	**UL[f]**	**Controls**	**Cases**	**OR[f]**	**LL[f]**	**UL[f]**	**Controls**	**Cases**	**OR[f]**	**LL[f]**	**UL[f]**	**Controls**	**Cases**	**OR[f]**	**LL[f]**	**UL[f]**
		**n= 2993**	**n= 286**				**n= 2821**	**n= 271**				**n= 1814**	**n= 165**				**n= 1814**	**n= 165**				**n= 1606**	**n= 154**			
**Total Meat**																										
Q1[g]	<3.0	749	43	1.00			707	38	1.00			461	23	1.00			461	23	1.00			409	23	1.00		
Q2[g]	3.0-4.2	748	58	1.31	0.87	1.97	700	55	1.28	0.82	1.99	455	33	1.23	0.70	2.17	455	33	1.23	0.70	2.18	397	30	1.11	0.62	1.99
Q3[g]	4.2-5.9	748	76	**1.72**	**1.16**	**2.54**	716	75	**1.58**	**1.02**	**2.42**	466	42	1.41	0.81	2.45	466	42	1.41	0.81	2.45	416	37	1.20	0.68	2.13
Q4[g]	>=5.9	748	109	**2.59**	**1.78**	**3.75**	698	103	**1.73**	**1.10**	**2.71**	432	67	**1.77**	**1.01**	**3.11**	432	67	**1.77**	**1.01**	**3.11**	384	64	1.66	0.94	2.95
P-trend				**<0.001**					**0.012**					**0.037**					**0.038**					0.064		
1 serving/week increase				**1.17**	**1.12**	**1.22**			**1.11**	**1.05**	**1.17**			**1.12**	**1.05**	**1.19**			**1.12**	**1.05**	**1.19**			**1.13**	**1.06**	**1.21**
**White Meat**																										
Q1[g]	<0.7	750	52	1.00			709	49	1.00			446	35	1.00			446	35	1.00			400	33	1.00		
Q2[g]	0.7-1.1	747	64	1.06	0.72	1.56	716	62	1.24	0.82	1.88	459	30	0.86	0.51	1.47	459	30	0.87	0.51	1.49	387	28	0.91	0.52	1.58
Q3[g]	1.1-1.5	748	78	1.15	0.79	1.68	698	72	1.05	0.70	1.57	461	42	0.84	0.51	1.38	461	42	0.85	0.52	1.40	411	37	0.80	0.48	1.35
Q4[g]	>=1.5	748	92	1.41	0.97	2.04	698	88	1.38	0.93	2.04	448	58	1.23	0.77	1.98	448	58	1.23	0.77	1.98	408	56	1.27	0.78	2.07
P-trend				**0.052**					0.195					0.349					0.353					0.336		
1 serving/week increase				**1.18**	**1.05**	**1.32**			1.11	0.98	1.26			1.09	0.94	1.27			1.09	0.94	1.27			1.10	0.94	1.28
**Red Meat**																										
Q1[g]	<1.1	749	45	1.00			710	39	1.00			459	26	1.00			459	26	1.00			406	24	1.00		
Q2[g]	1.1-1.8	748	61	1.27	0.84	1.90	700	58	1.39	0.89	2.15	447	32	1.23	0.70	2.14	447	32	1.22	0.70	2.14	399	31	1.30	0.73	2.31
Q3[g]	1.8-2.9	748	79	**1.60**	**1.07**	**2.37**	699	76	**1.73**	**1.12**	**2.66**	458	43	1.42	0.83	2.44	458	43	1.42	0.83	2.45	407	38	1.36	0.77	2.40
Q4[g]	>=2.9	748	101	**1.90**	**1.28**	**2.81**	712	98	**1.76**	**1.14**	**2.72**	450	64	1.56	0.91	2.66	450	64	1.55	0.91	2.66	394	61	1.62	0.92	2.83
P-trend				**0.001**					**0.009**					0.097					0.097					0.099		
1 serving/week increase				**1.16**	**1.08**	**1.25**			**1.11**	**1.02**	**1.20**			**1.13**	**1.03**	**1.25**			**1.13**	**1.03**	**1.25**			**1.14**	**1.03**	**1.26**
**Processed Meat**																										
Q1[g]	<1.4	749	46	1.00			700	43	1.00			463	28	1.00			463	28	1.00			411	26	1.00		
Q2[g]	1.4-2.6	748	64	1.35	0.90	2.01	706	59	1.25	0.82	1.92	474	32	1.03	0.59	1.77	474	32	1.02	0.59	1.76	424	31	1.04	0.59	1.83
Q3[g]	2.6-4.2	748	70	1.42	0.95	2.10	718	67	1.22	0.80	1.87	445	42	1.21	0.71	2.06	445	42	1.22	0.71	2.07	389	40	1.25	0.72	2.16
Q4[g]	>=4.2	748	106	**1.96**	**1.34**	**2.87**	697	102	1.48	0.97	2.28	432	63	1.45	0.85	2.48	432	63	1.46	0.85	2.49	382	57	1.40	0.80	2.45
P-trend				**0.001**					0.095					0.129					0.122					0.187		
1 serving/week increase				**1.07**	**1.03**	**1.10**			**1.04**	**1.00**	**1.08**			1.04	0.99	1.09			1.04	0.99	1.09			**1.06**	**1.00**	**1.12**

[a]Model 1: For total meat, models are adjusted by province of residence as a random effect. For white, red and processed meat, models are adjusted by other types of meat as fixed effects and province of residence as a random effect.

[b]Model 2: Model 1 +sex, age, education, family history of stomach cancer, physical activity (METs), smoking, BMI and energy, alcohol, fruits, salty fish and olives intake.

[c]Model 3: Model 2 restricted to individuals with complete information on HP infection.

[d]Model 4: Model 2+HP infection.

[e]Model 5: Model 2 restricted to individuals HP positive

[f] OR: Odds Ratio; LL: Lower limit of the 95% confidence interval; UL: Upper limit of the 95% confidence interval.

[g] Quartiles calculated among controls

Table S2. Sensitivity analysis to explore the role of *Helicobacter pylori* (HP) infection in the association between gastric adenocarcinoma incidence and meat-type specific doneness preference and cooking methods.

		**Model 1[a]**					**Model 2[b]**					**Model 3[c]**					**Model 4[d]**					**Model 5[e]**			
	**Controls**	**Cases**	**OR[f]**	**LL[f]**	**UL[f]**	**Controls**	**Cases**	**OR[f]**	**LL[f]**	**UL[f]**	**Controls**	**Cases**	**OR[f]**	**LL[f]**	**UL[f]**	**Controls**	**Cases**	**OR[f]**	**LL[f]**	**UL[f]**	**Controls**	**Cases**	**OR[f]**	**LL[f]**	**UL[f]**
**Donenes Preference**																									
**White Meat**	**n= 2878**	**n= 284**				**n= 2520**	**n= 244**				**n= 1622**	**n= 155**				**n= 1622**	**n= 155**				**n= 1436**	**n= 144**			
Rare/Medium	1825	153	1.00			1740	145	1.00			1079	93	1.00			1079	93	1.00			947	83	1.00		
Well-done	851	104	1.14	0.86	1.52	780	99	1.16	0.86	1.56	543	62	1.14	0.78	1.65	543	62	1.13	0.78	1.64	489	61	1.22	0.83	1.80
**Red Meat**	**n= 2910**	**n= 284**				**n= 2582**	**n= 253**				**n= 1660**	**n= 157**				**n= 1660**	**n= 157**				**n= 1470**	**n= 146**			
Rare/Medium	2180	191	1.00			2067	185	1.00			1304	109	1.00			1304	109	1.00			1145	99	1.00		
Well-done	564	75	1.24	0.92	1.67	515	68	1.23	0.89	1.69	356	48	**1.52**	**1.02**	**2.25**	356	48	**1.50**	**1.01**	**2.23**	325	47	**1.58**	**1.05**	**2.37**
**Cooking Methods**																									
**White Meat**	**n= 2878**	**n= 284**				**n= 2711**	**n= 269**				**n= 1736**	**n= 165**				**n= 2711**	**n= 269**				**n= 1540**	**n= 154**			
Griddle/BBQ	2072	193	**1.48**	**1.09**	**2.01**	1961	185	**1.49**	**1.08**	**2.07**	1196	115	**1.53**	**1.01**	**2.33**	1196	115	**1.53**	**1.00**	**2.32**	1058	105	1.37	0.89	2.11
Fried	1706	178	**1.48**	**1.13**	**1.95**	1625	172	1.30	0.98	1.74	1009	105	1.28	0.89	1.84	1009	105	1.28	0.89	1.85	886	99	1.36	0.93	1.98
Stewed	2205	241	**1.87**	**1.33**	**2.65**	2072	228	**1.71**	**1.19**	**2.47**	1313	139	**1.57**	**1.00**	**2.48**	1313	139	1.56	0.99	2.46	1168	131	**1.61**	**1.00**	**2.61**
Oven-Baked	1734	190	**1.83**	**1.37**	**2.44**	1654	179	**1.62**	**1.20**	**2.20**	1053	99	1.08	0.75	1.57	1053	99	1.08	0.75	1.56	935	90	0.98	0.66	1.43
**Red Meat**	**n= 2910**	**n= 284**				**n= 2740**	**n= 269**				**n= 1755**	**n= 164**				**n= 2740**	**n= 269**				**n= 1551**	**n= 153**			
Griddle/BBQ	2538	245	1.42	0.96	2.10	2395	237	**1.59**	**1.03**	**2.45**	1501	141	1.33	0.79	2.23	1501	141	1.34	0.79	2.24	1325	130	1.25	0.73	2.12
Fried	1900	204	**1.47**	**1.10**	**1.95**	1809	194	1.27	0.94	1.72	1142	117	1.25	0.85	1.84	1142	117	1.25	0.85	1.83	1004	112	1.35	0.91	2.03
Stewed	2532	261	**1.98**	**1.26**	**3.11**	2392	247	**1.62**	**1.01**	**2.60**	1525	150	1.53	0.85	2.76	1525	150	1.52	0.84	2.75	1350	139	1.36	0.75	2.48
Oven-Baked	1482	142	1.03	0.79	1.33	1418	136	0.95	0.72	1.25	902	81	0.84	0.59	1.19	902	81	0.83	0.58	1.18	805	77	0.84	0.59	1.22

[a]Model 1: For doneness preference, models are adjusted by type-specific meat intake as fixed effects and the province of residence as a random effect. For cooking methods, models are adjusted by other meat specific cooking methods as fixed effects and province of residence as a random effect and restricted to consumers of each type of meat.

[b]Model 2: Model 1 +sex, age, education, family history of stomach cancer, METS, smoking, BMI, energy, alcohol, fruits, salty fish and olives intake.

[c]Model 3: Model 2 restricted to individuals with complete information on HP infection.

[d]Model 4: Model 2+HP infection.

[e]Model 5: Model 2 restricted to individuals HP positive

[f] OR: Odds Ratio; LL: Lower limit of the 95% confidence interval; UL: Upper limit of the 95% confidence interval.
